# Applying Single-Cell Analysis to Gonadogenesis and DSDs (Disorders/Differences of Sex Development)

**DOI:** 10.3390/ijms21186614

**Published:** 2020-09-10

**Authors:** Martin A. Estermann, Craig A. Smith

**Affiliations:** Department of Anatomy and Developmental Biology, Monash Biomedicine Discovery Institute, Monash University, Clayton 3800, Victoria, Australia; martin.estermann@monash.edu

**Keywords:** DSD, sex differentiation, gonad, ovary, testis, single-cell, cancer, infertility, single-cell RNA sequencing, sex determination

## Abstract

The gonads are unique among the body’s organs in having a developmental choice: testis or ovary formation. Gonadal sex differentiation involves common progenitor cells that form either Sertoli and Leydig cells in the testis or granulosa and thecal cells in the ovary. Single-cell analysis is now shedding new light on how these cell lineages are specified and how they interact with the germline. Such studies are also providing new information on gonadal maturation, ageing and the somatic-germ cell niche. Furthermore, they have the potential to improve our understanding and diagnosis of Disorders/Differences of Sex Development (DSDs). DSDs occur when chromosomal, gonadal or anatomical sex are atypical. Despite major advances in recent years, most cases of DSD still cannot be explained at the molecular level. This presents a major pediatric concern. The emergence of single-cell genomics and transcriptomics now presents a novel avenue for DSD analysis, for both diagnosis and for understanding the molecular genetic etiology. Such -omics datasets have the potential to enhance our understanding of the cellular origins and pathogenesis of DSDs, as well as infertility and gonadal diseases such as cancer.

## 1. Introduction

Sex determination and sexual development in mammals can be divided into three broad stages. Firstly, chromosomal sex is established at fertilization, due to the differential inheritance of sex chromosomes. Oocytes typically carry a single X sex chromosome, whereas the sperm can carry an X or a Y chromosome, resulting in XX or XY embryos. This establishes chromosomal sex. Subsequently, during embryonic development, the undifferentiated gonad becomes either a testis (XY) or an ovary (XX). This process is known of gonadal sex differentiation is genetically regulated. The *SRY* gene in the mammalian Y chromosome activates the testicular differentiation program, whereas its absence in XX embryos allows ovarian differentiation [1,2,3,4,5,6]. Lastly, “somatic” sexual differentiation occurs, during which the gonads produce hormones that masculinize or feminize the external genitalia and the rest of the body. In eutherian mammals, testis-derived androgens induce formation of the male reproductive tract from the Wolffian duct, and Anti-Müllerian Hormone (AMH) induces regression of the Müllerian ducts. In females (XX), absence of fetal androgens and AMH results in regression of the Wolffian ducts and differentiation of the Müllerian ducts into the female reproductive tract. Externally, androgens induce formation of the penis and scrotum in XY individuals, whereas absence of androgen allows the external genitalia to become clitoris and labia in XX individuals [7]. Developmental variations at any of these stages can lead to Disorders (or Differences) of Sex Development (DSDs). These are congenital conditions in which development of chromosomal, gonadal or anatomical sex is atypical [8]. Gonads play a central role in translating the chromosomal and genetic signals into phenotypic sex. Genes involved in gonadal sex determination are often mutated in DSDs patients [9,10], demonstrating the importance of studying gonadal development to understand the causes of these conditions.

In recent years, the study of normal and abnormal gonadal development has moved into the so-called “-omics” era, whereby whole genome or whole transcriptome datasets have been analyzed. Such approaches are shedding new light on typical embryonic gonadal development, gonadal maturation, infertility, the somatic-germ cell niche, gonadal cancer and DSDs. DSD diagnosis has been advanced with the implementation of next-generation sequencing (NGS) methods such as whole exome and whole genome sequencing. This allows the detection of novel genes and regulatory regions affected in DSD [11,12]. While several Copy Number Variants (CNV) or single nucleotide polymorphisms (SNP) are detected per patient by NGS, it is difficult to determine the clinical relevance of such variance, requiring a better understanding of the exact genotype/phenotype correlation in DSD [10]. Functional studies in animal models have been very helpful in validating new DSD candidate genes [13,14,15], but this is time- and labor-intensive. Single-cell sequencing now has the potential to shed new light on DSDs, infertility and gonadal cancers. Rather than using the whole gonad as the source tissue, -omics data can be obtained at single-cell resolution. This technology allows genomic or transcriptomic changes within tissues to be assessed with granular detail, providing information of the different cell types present in normal and abnormal tissues. This review will focus on the application of emerging single-cell sequencing technologies to understand normal and atypical gonadal sex differentiation and function.

## 2. Gonadal Sex Differentiation

**Figure 1:**
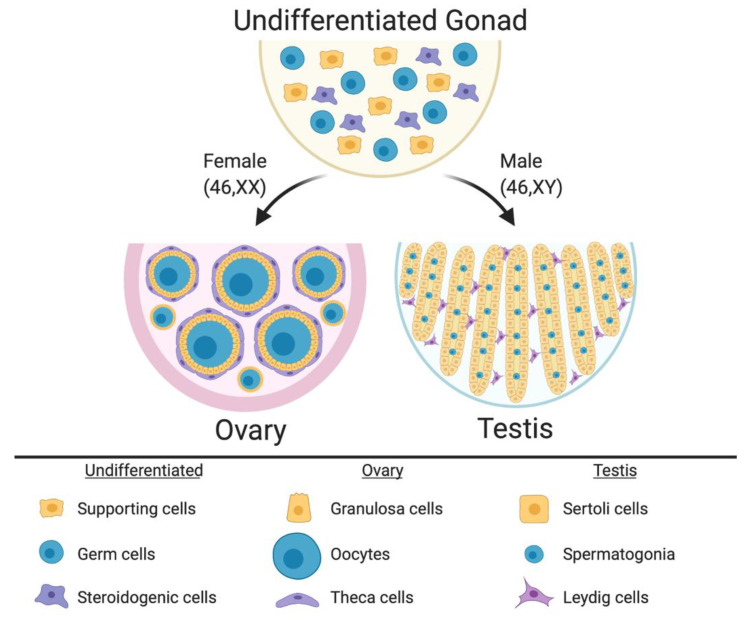
The undifferentiated mammalian gonad during embryonic life is morphologically identical between the sexes, comprising somatic cell precursors (steroidogenic and supporting cells) and germ cells. During sex differentiation, the gonadal cells types follow the ovarian or testicular pathways. In females, the supporting cells differentiate into pre-granulosa cells, which enclose the oocytes to form the primordial follicles. Pre-antral follicles comprise steroidogenic theca cells in the most external follicle layer. In males the supporting cells differentiate into Sertoli cells, which enclose the germ cells and form the testicular cords. Leydig steroidogenic cells being located in the interstitium around the cords. Image created with BioRender.com.

In mammalian embryos, the gonadal primordium is initially morphologically identical between the sexes. It comprises somatic cell precursors (future supporting cells and steroid hormone-producing cells) and germ cells (Figure 1) [16,17,18,19]. The gonad at this stage is considered “indifferent” or “bipotential.” The somatic cell progenitors follow either the testicular or ovarian pathway depending upon their sex chromosome constitution. In XY embryos, the *SRY* gene is activated, and this triggers differentiation of a progenitor subset into the Sertoli cells, which enclose germ cells and support their ultimate development into spermatogonia and spermatozoa. The Sertoli cells become arranged into testicular cords, lined by basement membrane (Figure 1). A subset of somatic cell precursors is subsequently directed to differentiate into steroidogenic fetal Leydig cells [20,21]. These cells, together with peri-tubular myoid cells, become distributed around the testicular cords [22,23,24,25,26,27]. While the Sertoli cells secrete Anti-Müllerian Hormone (AMH) to direct Müllerian induct regression, Sertoli and Leydig cells also synthesize androgens (testosterone) to direct masculinization of the Wolffian ducts and external genitalia [28]. In XX embryos, the same set of precursor cells follows a different fate. In the absence of *SRY*, autosomal genes such as *R-SPO1, WNT4* and *FOXL2* direct the ovarian pathway [29,30,31,32]. Homologous to the supporting Sertoli cell lineage [33], pre-granulosa cells differentiate around the germ cells, forming primordial follicles that arise in two successive waves [34] (Figure 1). Homologous to the Leydig cells, steroidogenic thecal cells differentiate around the follicles, a process that is not complete until after birth in mammals [35,36,37].

## 3. Disorders of Sex Development

**Figure 2:**
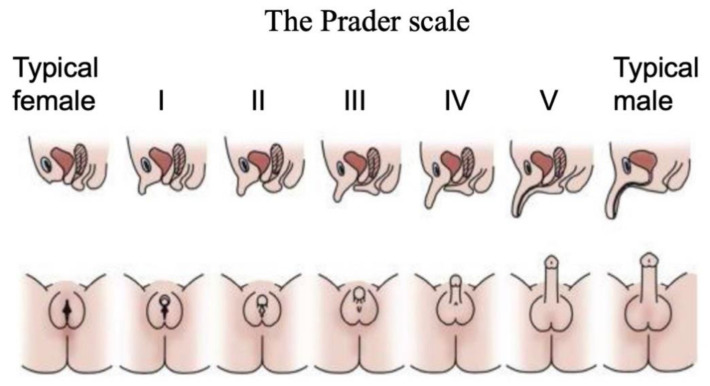
The Prader scale for external genital scoring. Typical female external genitalia are designated 0, and typical male at V or VI. Images above show cross sections, where the urinary and reproductive systems use separate orifices in females, a single orifice in males, and partially merged in intermediate cases. Images below show ambiguous external genitalia, graded between typical female (labia/clitoris; stage 0 or I) and typical male (scrotum/penis; stage V or VI). Adapted with permission from McNamara et al. (2017).

Disorders (or differences) of Sex Development are a major pediatric issue, comprising approximately 1% of all live births [38,39,40]. DSDs comprise a diverse range of conditions associated with atypical chromosomal, gonadal or anatomical sex [41]. They are typically diagnosed at birth or at puberty through external genital phenotyping, karyotype analysis, serum hormone measurements and targeted gene sequencing [42]. The Prader scale of external genital assessment has for many years been used as a coarse measure of initially diagnosing babies born with a DSD [43]. As in the case of the internal gonads, the external genitalia are initially morphologically identical between the sexes, comprising genital tubercle, genital fold and genital swellings. These become the penis and scrotum in males, and the clitoris and labia of females. On the Prader scale, typical female external genitalia are designated 0, and typical male at 5 or 6. Ambiguous external genitalia are graded between these two points (Figure 2). This scale reflects degree of external “masculinization” in a 46,XX baby or degree of “feminization” in a 46,XY baby.

Phenotypically, DSDs broadly fall into two categories; those due to atypical gonad formation and those due steroid hormone dysfunction (Table 1). In the first case, individuals are chromosomally 46,XX or 46,XY, but the gonads are “ambiguous”, sex-reversed or dysgenic (undifferentiated fibrous tissue). Individuals with 46,XY DSD at the gonadal level have gonadal dysgenesis and are externally “female”, due to an absence of testosterone synthesis and action. Approximately 45% of 46,XY DSD at the gonadal level can be attributed to loss-of-function mutations in the testis-inducing genes, *SRY* [3,44], *SOX9* [45,46] or upstream regulators of *SRY*, such as SF1, GATA4 and MAPK [47,48,49,50,51,52,53]. However, around 55% of 46,XY DSD as the gonadal level cannot be explained by mutations in known genes [48,54]. The 46,XY DSDs are highly associated with the incidence of gonadal tumors and infertility [55].

Individuals with 46,XX DSD attributable to a gonadal defect are genetically XX but have testes. Often first diagnosed at infertility clinics, these individuals have small testes, sterility, no female ducts and external genitalia that range from male to ambiguous [56]. The vast majority of 46,XX DSD cases at the gonadal level are due to aberrant translocation of the testis-determining gene, *SRY*, from the Y to the X chromosome during paternal meiosis [48]. Hence the entire male developmental pathway can be triggered, except for spermatogenesis. A very small percentage of 46,XX individuals have loss-of-function mutations in the ovary-promoting genes, *R-SPONDIN1* (*R-SPO1*) or *WNT4*, and around 9% have no known cause [57]. In humans with *R-SPO1* loss-of-function mutations, complete “sex reversal” can occur, whereby the gonads develop as testes despite the absence of the *SRY* gene [58]. These observations are consistent with the notion that SRY normally acts as “an inhibitor of an inhibitor” of testis formation and hence male development can proceed [59]. 

The second category of DSDs is involves aberrant hormonal signaling, downstream of gonadal sex differentiation. Among these, Androgen Insensitivity Syndrome (AIS) is the major cause of atypical sexual differentiation in individuals with 46,XY DSD [60]. Individuals with AIS express *SRY*, develop testes and secrete testosterone, but tissues are unresponsive (or are only partially responsive) to the hormone. [56]. Hence, individuals are “feminized”, or have ambiguous external genitalia (Prader scale 0–4) (Figure 2). Many cases of complete androgen Insensitivity (CAIS) are attributed to loss-of-function mutations in the Androgen Receptor (*AR*) gene [60,61,62]. However, *AR* mutations are less frequently reported in cases of partial AIS (PAIS) [62], pointing to other genes being involved. Another gene that can be mutated in 46,XY DSD at the hormonal level encodes 5-alpha-reductase (*SRD5A1*). This enzyme converts testosterone to dihydrotestosterone (DHT) in the external genitalia. Gonads develop as testes and release testosterone, which masculinizes internal ducts, but the external genitalia are variably feminized due to DHT deficiency [63,64]. 

The 45,XX DSD at the hormonal level is most commonly due to Congenital Adrenal Hyperplasia (CAH) [65]. These individuals, in fact, account for around 95% of all cases of 46,XX DSD. Typical ovaries develop but the adrenal gland aberrantly produces androgens during embryonic life, due to a loss-of-function mutation in the gene, *CYP21A2* [66]. This gene encodes 12-hydroxlase enzyme, part of the steroid hormone biosynthetic pathway. The 46,XX DSD CAH individuals have ambiguous or virilised external genitalia (Prader scale 4–6). They have relatively normal development of Müllerian structures and ovaries. In most cases, 46,XX DSD also causes infertility [67]. A rare subset of DSD is ovo-testicular disorder, in which both ovarian and testicular tissue can be present in the same individual [68] (Reviewed in detail in [69]). Lastly, sex chromosome aneuploidy has traditionally also been categorized as a DSD (so-called “chromosomal DSDs”). These include 46,XXY (Klinefelter Syndrome) and 46,XO (Turner Syndrome) [69]. However, in both cases, gonads and hormones are relatively normal and infertility is the main sexual phenotype. 

As noted above, DSDs are complex conditions, mainly due to the bipotential nature of the gonadal development, affecting both gonadal and extragonadal tissues [70]. Diagnosis and management of people with DSDs is complex, involving a multi-disciplinary team of geneticists, endocrinologists, genetic counsellors and family physicians [71]. In many cases in most countries, genital surgery has been conducted on newborns with DSD, such that genital sex matches chromosomal sex, and gonads are often removed due to the risk of cancer [72,73]. However, this has become a very controversial practice [74]. The decision to conduct genital surgery and gender assignment at birth is fraught with bioethical concerns centered around the patient’s rights to decide their own genital sex and gender identity [75,76]. In addition, DSDs can be induced by environmental rather than purely genetic factors, especially during gestation. Maternal luteomas, androgen-secreting tumors and exposure to exogenous androgen influence the development of 46,XX DSD [69]. Significantly, the majority of human DSD cases cannot be explained at the molecular level [12], which brings challenges to diagnosis and management [77].

## 4. Diagnosis of DSD

In newborns, DSD is typically diagnosed on the basis of external genital phenotype, in which ambiguous genitalia are noted. The so-called Prader scale, noted above, is used to coarsely assess external genitalia, with 0 being typical female, 6 being male, and ambiguous tissues being graded in between these values (Figure 2) [43]. Such analysis can now also be performed prenatally, at around 13–15 weeks [78]. Other tests include magnetic resonance and ultrasound of the reproductive tract and gonadal development, as well as endocrine assays [10]. In addition, cytogenetic tests and karyotyping are often used to detect abnormalities in chromosome number or macrostructure, identifying 46,XX or 46,XY DSD [12]. When karyotypic analysis is inconclusive, a more extensive approach is undertaken. CAH is diagnosed on the basis of biochemical screening. Elevated serum (or amniotic) levels of the precursor steroid, 17-hydroxy-progesterone (17-OHP) point to adrenal hyperplasia and excess androgen production in 46,XX individuals [66,79]. Furthermore, targeted sequencing of the *CYP21A2* gene can be conducted to detect mutations [10,80]. Other endocrine tests include Anti-Müllerian hormone, cortisol and gonadotropin measurements [70,81]. While they provide general information on the etiology of the DSD, biochemical assays alone are usually insufficient to determine the exact genetic cause. In the case of 46,XY DSD due to androgen insensitivity, levels of testosterone and Luteinzing Hormone (LH) can be elevated (although not always) [82]. In addition, hormonal assessment is helpful for monitoring precocious puberty and provides key information when designing hormonal therapy [83].

DSD diagnosis often also relies upon molecular genetic analysis, either to confirm a diagnosis or as the next step when endocrine tests are uninformative. Genes encoding key gonadal-sex determining genes can be assessed in the case of DSD at the “gonadal level”, while genes encoding steroidogenic enzyme or steroid receptors can be examined in DSDs at the “hormonal level” (Table 1). This can be time consuming and highly ineffective, resulting in the lack of a definitive diagnosis in a large number of patients [10]. Currently, the majority of DSD cases at the gonadal level have unknown molecular etiology [40]. Most recently, whole genome or whole exome sequencing has been adopted in an effort to diagnose and manage DSD patients where the molecular basis is not known [12,48]. However, it has been reported that 97% of undiagnosed patients in the American DSD Translational Research Network have not exhausted the available diagnostic testing clinically available, especially next generation sequencing methods [67]. Genetic panels have recently been developed as an economical alternative to next generation sequencing. These panels comprise genes known or suspected of being involved in DSD (around 30 genes). The genes are screened by next generation sequencing to identify causative mutations [40]. The panels can include coding and non-coding regions and regulatory elements [12]. Genetic panels provide a cost-effective test with the power to determine the genetic causes of DSD, showing around a 40% diagnostic rate [40]. While it allows for a rapid and cheaper genetic DSD diagnosis, this method relies on known genes and is not useful for identifying novel mutations related to DSD. 

For those cases that cannot be explained by mutations in known DSD genes, other methods are required to achieve an accurate diagnosis. Chromosomal microarrays provide a genome wide high-resolution scanning, allowing a rapid screening for genetic imbalance. Two main types of microarrays are used, comparative genomic hybridization (CGH) and SNP arrays [84,85]. CGH arrays compare the patient’s genomic DNA with a control by hybridization. Due to different fluorescently labelled samples, microduplications or microdeletions are detected, as well as copy number variants (CNVs). Duplications of known “sex genes”, for example, can underlie DSDs [86,87,88]. However, this technology does not identify translocations and inversions. In SNP arrays, an individual’s DNA is fluorescently labelled and hybridized to a genome representative array of oligonucleotides. Instead of comparing it with a control sample, as in the CGH, the SNP arrays compare the test sample with a pool of control samples available online. This technology can identify genetic gain or loss and, unlike the CGH array, is able to detect homozygosity, heterozygosity and consanguinity [11,89]. Parental samples are important to include in these approaches, to rule out inherited non-pathogenic polymorphisms.

With the reduction in the costs of next-generation sequencing and fast turnaround times, whole exome and genome sequencing have revolutionized the clinical testing for complex disorders, including DSD [10,11,12]. Whole exome sequencing allows the sequencing of approximately 95% of all the genomic protein coding regions. In addition to the protein coding regions, which represents a small portion of the human genome, whole genome sequencing provides information on the non-coding regions of the genome, including regulatory regions. The main caveat around these next-generation technologies centers on the large amount of information obtained. NGS identifies thousands of genome-wide variants, with the challenge being to distinguish pathogenic mutations [90]. To avoid this issue, trio analysis is usually performed, where the patient sequence data is compared with that of both unaffected parents. This identifies *de novo* mutations and reduces the number of variants to analyze [39,89].

All current methods used in diagnosing DSDs have limited potential to provide detailed phenotypic or functional information. Once the mutations and novel candidate genes are identified, validation of the effect in vitro or in vivo using animal models is a crucial, but time-consuming process. An exhaustive genotype/phenotype characterization will result in a complete understanding of the etiology, phenotype and associated risks with the DSD [89]. The identification of novel genetic alterations as well as genetic regulatory networks that underlie a specific DSD is crucial to understanding etiology and mechanism. Novel approaches will improve the diagnosis and management of DSDs [70]. Such approaches can include strategies of single-cell analysis.

## 5. Single-Cell Sequencing Technologies

**Figure 3:**
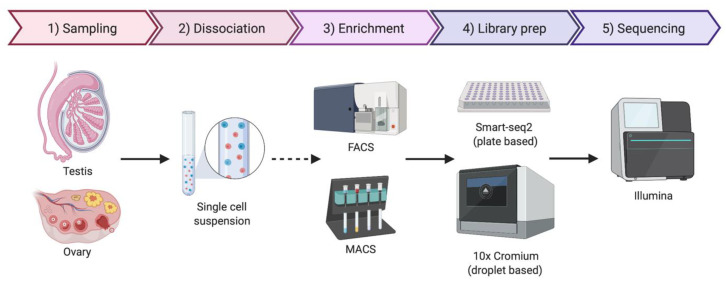
Simplified protocol for single-cell transcriptomics or genomics, from the sample collection to sequencing. Briefly, the tissue sample is dissociated to obtain a single-cell suspension. An optional enrichment/sorting step could be required for rare cell types (dashed arrow). cDNA libraries are then prepared, barcoded to identify individual cells and individual transcripts and sequenced. If the gonadal cellular composition is more important, then 10× Chromium technologies if often chosen. If sequencing depth is more important, Smart-seq2 is typically preferred. Image created with BioRender.com.

As the name implies, single-cell sequencing provides genomic or transcriptomic data at single-cell resolution. This approach has the potential to shed new light on the biology of gonadal development, infertility, gonadal cancer and DSDs. Previously, cell purification was required to gain insight into cell type-specific genetic programs. This has typically required the use of specific cell surface markers and fluorescence-activated cell sorting (FACS) followed by RNA-seq [91]. Transgenic mice expressing cell-specific fluorescent proteins are also widely used for specific cell-type sorting. However, this is a time-consuming method and requires knowledge of gene regulatory regions active only in the cell type of interest [92]. Single-cell transcriptomics provide gene expression information at single-cell resolution and, in contrast to the previous sorting methods, it does not require any *a priori* knowledge. By this methodology, transcripts in individual cells are genetically bar-coded and sequenced, allowing the bioinformatic clustering of cells based on shared transcripts (Figure 3) [93]. This method allows cell lineages to be characterized and traced within a tissue or organ [94]. The method can also detect the emergence of aberrant cell types, in the case of cancer and other diseases [95]. Single-cell technologies require the purification of individual cells from tissue samples, typically by mechanical or enzymatic treatment, to generate a single-cell suspension [96]. Cell isolation can be achieved using diverse methods, including micro-pipetting, magnetic activated cell sorting (MACS) and FACS (Figure 3). With the incorporation of microfluidic technologies, this process has become semi-automated, allowing more reproducible results and avoiding DNA contamination [96,97]. Two main technologies are generally used for single-cell RNA sequencing (scRNA-seq), the droplet based 10× Genomics Chromium platform and the plate based Smart-seq (Figure 3) [98,99,100]. Smart-seq2 is more sensitive for transcript detection (especially low levels of expression), whereas 10× captures more cells, allowing superior detection of rare cell populations [99]. The two methods can to be complementary, so the choice of one or other methodology depends upon research aims.

**Figure 4:**
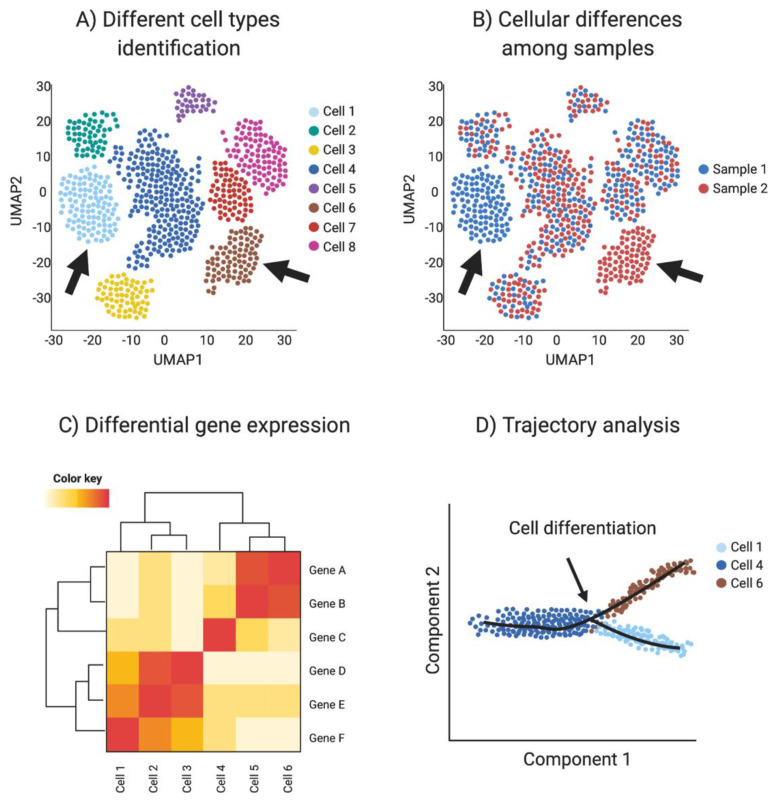
Single-cell RNAseq (scRNA-seq) analysis. (**A**) Spatial reduction analysis and clustering based on shared transcripts identifies different cell types present in a sample. Data is represented as UMAP plots (Uniform Manifold Approximation and Projection), an algorithm for visualizing high dimensional data in a two-dimensional space, such that similar objects are grouped. The data can be interrogated for individual gene transcripts, showing the cell type/s of expression. (**B**) scRNA-seq can be used to identify sample-specific cell types when comparing two or more different samples, for example when comparing ovaries and testis or mutants with wild type (red vs. blue in this example, where arrows show tissue-specific cell types). (**C**) Single-cell transcriptomics can be used to detect differential gene expression among cell types and samples to identify novel cell specific markers (Red in this example). (**D**) Trajectory analysis (pseudotime) uses transcriptomic information of each cell to provide information regarding cell differentiation, including how and when cell lineages emerge and diversify. Image created with BioRender.com.

Single-cell RNA-seq provides transcriptomic information for every sequenced cell, allowing the detection of small transcriptome changes. Using these highly variable genes, cells are plotted in a bi-dimensional space, such that similar cells are grouped. One of the most common methods for high dimensional data vitalization used in single-cell RNA sequencing is the t-Distributed Stochastic Neighbor Embedding (t-SNE) [101]. Another dimensionality reduction algorithm used in single-cell data is uniform manifold approximation and projection (UMAP). UMAP preserves the local and global structure better than the t-SNE and is faster to run [101], resulting in a preference in the single-cell community towards UPAM algorithm. Clustering techniques are used to identify different cell types or clusters in the dataset, based in their shared transcriptome. In addition, transcriptomic information can be used to detect differential gene expression among cell types and samples to identify novel cell specific markers. (Figure 4A–C). 

Cell differentiation involves transcriptional changes through a series of intermediate states. Trajectory analysis (pseudotime) uses transcriptomic data of each cell to provide information regarding cell differentiation, including how and when cell lineages emerge and diversify. Moreover, novel intermediate states and molecular triggers for these cell fate specification events can be detected by so-called pseudotime analysis (Figure 4D). The preservation of the global and local structure by this method is crucial to provide a precise trajectory inference, and is the reason why UMAP is the preferred dimensionality reduction algorithm used in this kind of analysis. In addition, pseudotime analysis can be enriched by incorporating real time events, for example using a developmental series to study cell differentiation in real time [93,102]. 

In addition to single-cell transcriptomics, other-omics data can be gathered at the single-cell level. Single-cell genomics can be applied to CNV as well as SNP, the latter requiring a higher sequencing depth [103]. Single-cell DNA sequencing has been used to broadly study tumor cell composition and to understand the effects of genetic mosaicism [102]. Among the epigenetic analyses available, single-cell ATAC-seq and single-cell DNA methylome have been developed to identify chromatin accessibility and DNA methylation with single-cell resolution [104,105]. Despite these diverse applications, the most well- developed single-cell technology is presently scRNA-seq [103]. 

**Figure 5:**
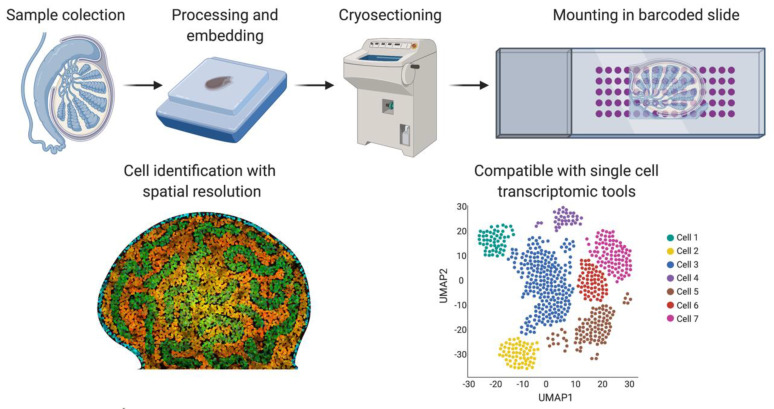
Spatial transcriptomics. Briefly, fresh frozen tissue is cryo-sectioned and mounted into a slide containing barcoded spots that provide a specific spatial orientation. The cells are permeabilized and the mRNA binds to the most proximal barcode, correlating single-cell transcriptomics with a spatial tissue location. This technique is also compatible with the different sc-RNA-seq analysis shown in Figure 4. Image created with BioRender.com.

One of the main issues around single-cell technologies is that spatial and tissue organization information is lost when generating a single-cell suspension. Spatial transcriptomics [106] was developed as a new methodology to overcome this problem (Figure 5). Frozen tissue sections are placed on a slide containing barcoded spots that provide a specific spatial orientation. The cells in the tissue sections are permeabilized, allowing mRNA to be bound to the most proximal barcode, followed by cDNA synthesis and sequencing. The barcode sequence associates each transcript with a spatial position, assigning mRNA expression to different tissue regions [102]. A negative feature of this technique is that the barcoded spots are not small enough to be representative of a single-cell, but refinements have been achieved in recent years. We can expect a higher number of smaller barcodes in the future to achieve spatial transcriptomics of single-cells.

In the need for improved genotype/phenotype correlation, single-cell multiomics have been generated [103]. The most popular are combinations of scRNA-seq with single-cell genomics, DNA methylation or ATAC-seq for regulatory regions. This methodology relies on the different localization patterns of the nucleic acid within a cell, mRNA in the cytosol and DNA in the nucleus [107]. Selective cell membrane lysis allows the separation of the mRNA from nuclear DNA, which remains intact and can be later isolated by centrifugation or via antibody conjugated magnetic beads, prior to cell lysis [108]. 

Nucleic acids are not the only focus of the single-cell multiomics. CITE-seq (cellular indexing of transcriptomes and epitopes by sequencing) is a method that combines the use of antibodies to detect different proteins with scRNA-seq [109]. While it is most applicable to surface markers, this methodology, in theory, can be applied to any known protein. The main limitation here is the availability of high-fidelity antibodies [102]. The method uses streptavidin-biotin interaction to bind the 5′ end of an oligo to antibodies, to barcode the antibodies. The cell suspension is incubated with as many antibodies as desired, and the single-cell RNA sequencing is performed (10× Chromium) [109]. Levels of protein expression and transcriptomics are simultaneously measured for each sequenced cell, matching the proteome and the transcriptome. This is particularly helpful when specific protein markers are associated with different cell states, which is key to define immunological cell states, for example [102].

Cell Hashing [110] uses a similar approach to CITE-seq, but instead of labelling specific cell types or specific proteins, it uses an antibody against a ubiquitously expressed cell surface protein to label an entire sample. Labelling each sample with an antibody containing a specific barcode allows the samples to be pooled and processed together to generate a unique library, reducing the costs of the run and reducing the batch effect [110]. Due to each sample being labelled with different barcodes, each sequenced mRNA will have a sample and a cell identifier.

Single-cell sequencing shows potential as a novel technological platform for biomedical research and diagnosis, especially in complex diseases [96,103]. The minimal sample requirement, together with the ability to detect protein, DNA and mRNA simultaneously, positions single-cell multi-omics as a promising technique for translational medicine. Single-cell -omics can potentially provide a precise relationship between the genotype and phenotype, a missing feature in many current DSD, infertility and gonadal cancer diagnosis methods.

In order to use single-cell sequencing in the clinic as a tool for diagnosing DSD, it is necessary to firstly generate testicular and ovarian atlases from embryonic development through adulthood. The Human Cell Atlas [111] seeks to generate a collection of organ specific reference maps at single-cell resolution. These atlases can be useful to identify cellular and molecular changes associated with different diseases. While some organ-specific atlases have been published [112,113], complete ovarian and testicular human atlases from embryonic development through adulthood are yet to be developed. Several groups have been studying gonadal development, differentiation and maturation at the single-cell level in a wide range of organisms (Table 2). Great efforts have been made to generate gonadal databases with high diagnostic value, resulting in the development of an adult [114] and pubertal [115] human testicular atlas. This demonstrates the potential of this technique in dissecting a complex developmental process.

## 6. Single-Cell -Omics in Gonadal Development and Maturation

### 6.1. Early Gonadal Development and Differentiation at the Single-Cell Level

As described above, chromosomal sex is determined at fertilization, when the haploid genomes of sperm and egg fuse to generate a diploid zygote. In mammals, depending on the sex chromosome carried by the sperm, the resulting zygotes can be 46,XX (female) or 46,XY (male) [141]. While gonadal sex is determined later in embryonic development, single-cell sequencing data from early embryos (4-cell to late blastocyst), show that male and female human embryos exhibit differential gene expression soon after embryonic genome activation, well before gonadal development [142]. *RPS4Y1* and *DDX3Y* genes from the Y chromosome are highly expressed in male embryos, for example, whereas in females X chromosome genes such as *HNRNPH2* and *DDX3X* escape chromosome dosage inactivation and are expressed from both X chromosomes simultaneously [142]. 

Ovaries and testis are dynamic organs that function to producing gametes and sex hormones, both key to proper sexual reproduction. Both organs, although anatomically and histologically different, derive from a common precursor (Figure 1). During embryonic stages in mammals, the gonadal ridge develops as a thickening of the coelomic epithelium, on the ventromedial surface of the mesonephric kidney, marking the formation of the gonadal primordium [18]. Coordinated proliferation of the coelomic epithelial cells, delamination and ingression of those cells results in the further development of the primordium [143]. In mammals, ingressing cells are the undifferentiated precursors of the supporting and steroidogenic cell lineages (Figure 1) [17]. In addition, other cell types migrate into the genital ridge from the mesonephric kidney, namely, endothelial and interstitial cells [144]. Primordial germ cells (PGC’s) are specified in the epiblast and migrate into the gonadal ridges through the hindgut and mesentery [18,145]. Single-cell RNA-seq has been used to characterize the diverse gonadal populations in human, mouse and chicken embryos [21,116,119,133,134]. In all species analyzed, somatic and germ cells from males and female gonads show no major gene expression differences prior to gonadal sex differentiation, apart from dosage-related sex-linked genes. Specific cell types are equally detected in both sexes, consistent with the undifferentiated and bipotential character of gonads at these early stages.

The undifferentiated gonads become sexually determined around embryonic day (E) 11.5 in mouse and gestational week 6 in humans. Sex chromosomes play a key role in this process, notably the *Sry* gene present in the Y chromosome [146,147]. In the mouse, *Sry* becomes active in the supporting cells during a short period of time and is inactivated after the Sertoli cell program is initiated [35,49]. *SRY* encodes a master transcription factor. One of its major roles is activation of a related gene called *SOX9*. *SOX9* is a critical factor involved in activating the Sertoli cell genetic program, as well as repressing the female pre-granulosa pathway [148]. *SOX9* is necessary and sufficient to induce Sertoli cell specification [149,150,151]. After Sertoli cells are specified, they channel differentiation of the gonadal cell types into the testicular pathway. PGCs differentiate into sperm cells, steroidogenic cells into fetal Leydig cells and interstitial cells into peritubular myoid cells (Figure 1). In XX embryos, due to the absence of Sry, the WNT4/RSPO1/β-catenin pathway is stabilized, marking the first signs of pre-granulosa and ovarian differentiation (Figure 1) [152,153]. 

Single-cell RNA-seq has been used to identify sex specific cells clusters in human, mouse and chicken embryos at the onset of gonadal sexual differentiation. In mouse embryos, scRNA-seq was used to follow the fate of sorted *Nr5a1*+ cells, in both sexes. This gene encodes SF1 (Steroidogenic factor 1) expressed in most (if not all) early gonadal somatic cell progenitors before sexual differentiation. The data showed a clear developmental trajectory whereby these cells give rise to the supporting and then interstitial steroidogenic cells, in both sexes [133]. Before sex differentiation, the early progenitor cells commit to the supporting or the interstitial fate. By E12.5, supporting cells are fully committed to either pre-granulosa or Sertoli cell fate in the mouse. Interestingly, prenatal pre-granulosa cells and undifferentiated male supporting cells cluster together transcriptionally, suggesting shared transcriptomes and supporting their inferred homology and common origin. However, embryonic Sertoli cells cluster separately, suggesting a rapid downregulation of progenitor genes and upregulation of thousands of Sertoli specific genes, essentially triggered by the upregulation of *Sry* in males [21]. This research in the mouse model focused in a specific somatic progenitor lineage, so the transcriptomics of other male gonadal cell types that are not derived from *Nr5a1*+ cells, such as germ cells, have not yet been reported. 

In females, single-cell RNA sequencing using whole gonadal ovarian tissue has been performed at different developmental timepoints, ranging from E11.5 to postnatal day (P)5 [34]. These timepoints permitted a continuous analysis of different stages of folliculogenesis, from primordial germ cell sex determination, mitosis, meiosis, pachytene arrest and formation of primordial follicles. Germ cells were identified in pre-meiotic, pre-leptotene, leptotene, zygotene, pachytene, diplotene and dictyate stages [34]. This analysis provided unprecedented molecular detail on embryonic oogenesis, identifying stage specific genes for subsequent analysis. In addition to the germ cells, epithelial, mesenchymal and endothelial/blood related cells were identified in female gonads. Two pre-granulosa cells types were detected, a *Foxl2*^+^ “bipotential pre-granulosa cells” that derive from the supporting bipotential precursors and a *Lgr5*^+^ “epithelial pre-granulosa cells” that derive from the ovarian surface epithelium [34]. The former are associated with medullar follicles and the later with cortical follicles, consistent with previous reports [34,154,155]. The epithelial pre-granulosa population was not found in the ovarian *Nr5a1*+ sorted cell analysis described above [133], consistent with an epithelial origin. This emphasizes the utility of single-cell -omics using whole tissues as opposed to sorted cells.

Single-cell transcriptomics has been performed on whole human embryonic gonads, encompassing both somatic and germ cells [116,119]. In the human gonads, both Sertoli and pre-granulosa cells have been identified as independent clusters. In addition, Leydig or steroidogenic cells, macrophages and T cells were also detected [119]. As expected, germ cells were also sexually dimorphic, being arrested in mitosis in males and meiotically arrested in females [123]. Novel surface markers were identified for the different types of germ cells, useful for isolating fetal germ cells in meiotic prophase (IL13RA2^+^) and oogenesis (PECAM1^+^) [119]. This demonstrates the potential of single-cell transcriptomics to identify new cell specific markers. Single-cell transcriptomics has been very useful in shedding new light on the somatic-germ cell niche. In both sexes, a highly specialized microenvironment, or niche, exists that fosters proper development on the germline, through signaling from the supporting somatic cells (Sertoli cells in males and granulosa cells in females). Li and colleagues [119] interrogated the transcriptomes of over 2000 fetal germ cells and their gonadal niche cells from male and female human embryos across many developmental stages, using single-cell RNA-seq analysis. Their study found that human female fetal germ cells transition through four distinct phases; mitosis, retinoic acid responsiveness, meiotic prophase and oogenesis. Male germ cells went through a migration phase followed by mitosis and cell cycle arrest. Cells in individuals embryos could be detected at any of these stages, highlighting the asynchronous nature of fetal germ cell development, while germ-somatic reciprocal BMP and Notch signaling pathways were enriched in the transcriptomic datasets [119]. 

Single-cell transcriptomics across different species has revealed different patterns of lineage allocation during gonadal sex differentiation. Birds for example, have a ZZ male; ZW female sex chromosome system, although the same gonadal sex types are present as observed in mammals [156,157,158]. However, unlike in mouse, the coelomic epithelium gives rise to the gonadal epithelium/cortex and the interstitial cells, but not the supporting cell lineage in chicken. Rather, the critical supporting cell lineages (Sertoli/pre-granulosa cells) derive from a mesenchymal population that expresses the transcription factors, *DMRT1*, *PAX2* and *OSR1* [134]. In addition, steroidogenic cells in chicken differentiate from the previously differentiated supporting cell lineage by a sequential up-regulation of the steroidogenic genes such as *CYP17A1* and *STAR*, followed by a down-regulation of the supporting cell markers [134]. Sexually dimorphic cell populations were also identified among the (non-steroidogenic) interstitial cells, and among epithelial and germ cells [134,159]. This research indicates that cell lineage specification in the gonad can vary from species to species. 

Gonadal differentiation relies upon mutually exclusive sexually dimorphic pathways that reinforce one genetic program and repress the other. In the recent years, it has been proposed that these two gonadal programs (ovary vs. testis) must be actively maintained throughout life. This prolonged mutual antagonism has been shown by bulk and single-cell RNA sequencing [118]. These studies have revealed that postnatal inactivation of the testicular genes, *DMRT1* and *SOX9* can lead to “trans-differentiation” of Sertoli cells to a granulosa-like cell fate [151,160]. Conversely, loss of the granulosa regulatory gene, *FOXL2*, can induce trans-differentiation of the gonad from ovary to testis [161]. These findings underscore the persistent mutual inhibition of the testicular and ovarian developmental pathways, governed by the somatic cell populations.

### 6.2. Testicular Maturation and Single-Cell Transcriptomics

While the testis is fully developed at birth, it becomes reproductively active postnatally, resulting in reactivation of quiescent germ cells. Spermatogenesis is a controlled process that begins postnatally and culminates with the production of the first sperm at the onset of sexual maturity (7 weeks in mice and 12–13 years in humans) [162]. This process is known as the first wave of spermatogenesis and involves a sequential differentiation of the primary spermatocytes through spermatogenesis and spermiogenesis. Because this first round of differentiation occurs during postnatal development, it is possible to capture all the germ cell stages and types by sampling different individuals through different developmental stages and generating a spermatogenesis atlas. A developmental time series of the mouse postnatal testis has been analyzed by single-cell RNA sequencing in order to elucidate the transcriptomic regulation of male gametogenesis [126,128]. The stages ranged from P5 to P35, allowing a temporal and cellular reconstruction of the developmental process of great diagnostic value, for example, in diagnosing types of infertility. In addition to germ cells, somatic cells were also analyzed during postnatal development, identifying fetal and adult Leydig cells, Sertoli cells, peritubular myoid cells and macrophages and genetic programs therein [126,137]. 

Single-cell RNA-seq has also been performed on the adult mouse testis, using sorted cells or whole testis and using different sequencing platforms [120,121,122,131,140]. A large number of germ cells were detected, in comparison with the somatic counterpart, consistent with the robust generation of spermatozoa in the seminiferous tubules. Most of these analyses have focused on the germ cells, detecting a continuum of 12–20 different germ cell states, significantly more than previously supposed [121,122]. These results are consistent with the process of spermatogenesis and spermiogenesis, demonstrating the continuous nature of germ cell differentiation and providing stage specific markers. This information will be useful for further isolation and characterization of normal and aberrant male gamete production.

Somatic cells identified by these analyses included macrophages, Sertoli, myoid, Leydig and endothelial cells, and newly reported populations, such as an innate lymphoid type 2 cell (ILCII) and a mesenchymal cell population that expressed high levels of *Col1A1, Tcf21* and *Arx*, but no *Acta2, Myh11* or *Cyp17a1* [121]. In addition, *Tcf21* positive cells were detected surrounding the seminiferous tubules and in the interstitial space [121]. This interstitial population was similar to the interstitial population reported in male mouse embryonic gonads [21], reported to give rise to the fetal Leydig cells. Four different Sertoli cell clusters were also identified that could be separated into nine different subtypes that correlated with the different seminiferous tubule stages. This suggests that there are functionally distinct Sertoli cell subtypes that vary across the different stages of seminiferous tubule maturation [121].

Most current knowledge of spermatogenesis is based upon non-human mammalian models, primarily mice and rats. Macaques are useful primate models, phylogenetically related and physiologically similar to humans [135]. In this regard, such primates represent an advantage over rodents and other model species [163]. Unlike in humans, sample collection in differential stages of development to generate a gonadal atlas can be readily achieved for non-human primates. Single-cell RNA-seq of testicular samples has been performed in cynomolgus [135] and rhesus [136] macaques. Germ cells, Sertoli, Leydig, myoid and endothelial cells were identified in both macaque testicular samples. In addition, macrophages, T cells and pericytes were identified in rhesus. This newly generated data has been compared with previous mouse and human analysis to identify conserved and non-conserved mechanisms in spermatogenesis. Endothelial and immune cells are highly conserved among species, whereas testis interstitial cells showed the greatest interspecific divergence, in terms of gene expression [136]. Germ cells from all three species showed a continuous trajectory of differentiation from spermatogenesis to the end of spermiogenesis [135].

Primate and rodent spermatogenesis are known to be significantly different, demonstrating the necessity of a human-specific testicular atlas. Having a complete time series of human gonadal development may seem challenging, but in recent years, single-cell RNA-seq from neonatal [132], pre, peri and post puberty [115] human gonads has been performed. Results demonstrate the utility of this scRNA-seq approaches for finely dissecting aspects of human reproductive development. Five major cell types were identified in human neonatal testis (2 and 7 days old): germ cells, peritubular myoid cells, Sertoli cells, Leydig cells and erythrocytes. Among the germ cells, two subsets were identified, one with a transcriptome similar to the embryonic primordial germ cells and the other expressing several pre-spermatogonia cell markers, denoting activation of meiosis and the start of spermatogenesis [132]. Whole human testis from 7, 11, 13 and 14 year-old (yo) male donors were also subjected to single-cell RNA-seq analysis [115]. Different cell types were also identified: germ cells (comprising spermatogonia, spermatocytes and post-meiotic spermatids), Sertoli, Leydig and smooth muscle cells, macrophages, myoid and endothelial cells. The germ cells contained slowly self-renewing and undifferentiated spermatogonia, differentiating spermatogonia (11 yo onwards), spermatocytes and spermatids. Fourteen-year-old germ cell composition was similar to that of adults, indicating complete spermatogenesis. In contrast, the 7 yo sample only contained undifferentiated spermatogonia. Sertoli cells also showed two immature and one mature state. During maturation, AMH expression levels decreased whereas androgen receptor was upregulated. This maturation was found to be gradual but asynchronous among different tubules [115]. Interestingly, the immature state 2 shows lower levels of expression of mitochondrial genes, a feature that was also reported in a novel chicken embryonic Sertoli cell population [134]. 

Single-cell RNA-seq has been performed on adult human testes, ranging from 17 to 60 years of age [114,122,124,132,136]. As in mouse, most research has focused on germ cells, where researchers identified around 8–13 different germ cell clusters that formed a continuum, resembling the spermatogenesis and spermiogenesis process. In addition, several somatic cells were identified, namely, macrophages, endothelial cells, peritubular myoid cells, Sertoli cells, Leydig cells, T cells, and two novel population that were considered pericytes (multi-functional cells of the microvasculature). One of the pericyte population expressed smooth muscle markers (m-pericytes) and resembled myoid and immature Leydig cells, whereas the other was highly enriched for extracellular matrix transcripts (f-pericytes), and resembled fibroblastic cells [136]. Interestingly, a population similar to the m-pericytes was identified in adult mouse testis (labelled as interstitial cells), suggesting conservation in mammals [121]. In summary, single-cell -omics has been instructive for understanding the cellular complexity of the testis, previously missed by bulk RNA-seq analysis. Altogether, scRNA-seq data from the embryonic and postnatal testis point to greater developmental complexity among somatic and germ cells populations than previously appreciated. 

### 6.3. Ovarian Maturation and Single-Cell Transcriptomics

The ovary is one of the most dynamic organs in the body, undergoing monthly organ wide remodeling to achieve follicle maturation, ovulation and corpus luteum formation or atresia [127,138]. During the second half of embryonic development in humans and immediately after birth in mouse, the primordial follicles form, consisting of a single oocyte surrounded by one layer of squamous granulosa cells [164]. Most of these primordial follicles die, while the remaining will form the ovarian reserve of follicles that continue follicular development. The transition from primordial to primary follicle is characterized by granulosa cells changing morphology from squamous to cuboidal [165]. Primary follicles transition into secondary follicles, containing two or more layers of granulosa cells and preantral follicles, containing steroidogenic theca cells in the most external follicle layer. Antral follicles are characterized by oocytes surrounded by two distinctive granulosa cell subpopulations, the cumulus cells (adjacent to the oocyte) and the mural granulosa cells. Hormones secreted from the pituitary gland are important during antral formation and especially during the transformation of antral follicles, preovulatory development and later oocyte release [164]. After ovulation, the remaining granulosa and theca cells form the corpus luteum [164]. Typically, only one oocyte is ovulated, whereas other antral follicles will degenerate, forming atretic follicles.

As for studies on testis development and maturation, RNA-seq has been applied to ovarian function. However, there have been fewer reported studies focusing on the ovary compared to the testis. One of the first studies was performed on different follicles in human ovarian tissue [125]. From each follicle, the oocyte and 10 randomly selected granulosa cells were subjected to scRNA-seq. Transcriptionally, oocytes were found to form five distinctive subpopulations, which correspond to the classification of follicular development (primordial, primary, secondary, preovulatory and antral) [125]. Interestingly, granulosa cell clusters from primary and secondary follicles overlapped transcriptionally, indicating a similar transcriptome. In a cortical ovarian single-cell RNA sequencing study, other somatic cells were also identified, such as endothelial cells, smooth muscle cells, three theca cell populations (healthy follicles, small antral follicles and early atretic), stromal cells and immune cells (monocytes/macrophages, T cells, NK cells and B cells) [127,138]. No oogonial stem cells were found in these analyses, and indeed the traditional germ cell marker, *DDX4*, does not identify any such cells. Instead, it detects cortical perivascular cells expressing smooth muscle and pericyte markers [138]. This is an interesting point, as the potential existence of oogonial stem cells has long been debated in the field of female reproductive biology [138,166,167,168]. Single-cell RNA-seq is therefore shedding new light on this debate. Given its exquisite sensitivity, single-cell transcriptomics so far does not support the notion of oogonial stem cells in the human adult ovary [138]. 

During adulthood, the ovarian reserve is reduced with each ovulation, culminating in the menopause around 50 years of age in humans. In addition to the decrease in the follicle number, the quality of the oocytes also is reduced with age, resulting in a decline in fecundity and an increase in chromosomal anomalies [169]. In addition to age, other factors can deplete the ovarian reserve, resulting in an early menopause. These factors include smoking, cancer, environmental pollutants and chemotherapeutic drugs [170]. Understanding the mechanisms of the ovarian ageing is crucial for predicting and preventing ovarian loss and extending fertility among women. Ovarian ageing has been studied using single-cell RNA sequencing in juvenile (4–5 yo) and aged (18–20 yo) cynomolgus monkeys [139]. Each cell type cluster comprised both juvenile and aged cells, suggesting that the cell type transcriptional differences were more pronounced than age-related changes. Seven different cell populations were identified, including oocytes, granulosa cells, stromal cells, smooth muscle cells, endothelial cells and immune cells (Natural Killer T cells and macrophages) [139]. Oocyte sub-clustering detected four different oocyte subtypes: primordial, primary, secondary and antral. Aged oocytes showed a reduction of antioxidant gene expression, contributing to the increment of oxidative damage during ovarian ageing [139]. Difference was also evident among the granulosa cells, showing increased apoptosis and reduced reductase activity, which could also explain the increase of oxidative damage [139]. Due to the difficulty in obtaining human ovarian tissue, the cynomolgus monkey is a promising model for studying ovarian ageing. As for testis biology, ovarian scRNA-seq provides new insights in the complex process of functional maturation and ageing. This information will significantly enhance our understanding of infertility and gonadal cancer. It will also inform methods of fertility preservation and assist in identifying early markers of gonadal ageing.

## 7. Single-Cell -Omics in Gonadal Disease

While single-cell transcriptomics is not routinely available in the clinic, several research groups have used these technologies to study a wide range of gonadal conditions, including DSDs, infertility and cancer, with informative results (Table 3). 

### 7.1. DSDs

Single-cell sequencing approaches have the potential to inform DSDs and sex chromosome aneuploidies, although there are currently few documented. One study focused on Klinefelter Syndrome (47,XXY), which results in tall-statured males with infertility (two X chromosomes interfere with spermatogenesis) [180]. Single-cell RNA sequencing has been performed on 47,XXY peripheral blood monocytes (PBMCs) to investigate the effect of supernumerary sex chromosomes on the immune system at single-cell resolution [176]. This is the first and to date only scRNA sequencing analysis of Klinefelter syndrome, and also the first in the context of a “chromosomal DSD”. The results were compared with typical male and a female PBMCs samples, allowing identification of T cells, Natural Killer cells, B cells, macrophages and dendritic cells. Patient cells transcriptionally clustered together with the healthy male and female donor cells. Moreover, the proportions of cell types in the patient were similar to the male donor, but statistically different to the female, suggesting a male like phenotype [176]. In addition, transcripts from both X chromosomes were identified in different cells, expressing *XIST* transcript at similar levels to female samples. This suggested that the additional X chromosome in the Klinefelter syndrome patient was inactivated. Interestingly, most of the differentially expressed genes between the patient and the healthy donors were autosomal genes [176]. Further studies will be needed to identify how the additional X chromosome can affect the expression of autosomal genes and specifically which X linked genes can escape inactivation. The gonadal composition of the Klinefelter syndrome at a single-cell resolution is yet to be explored.

Single-cell RNA-seq has not yet been applied to the field of DSD but has been used to examine the effects of hormonal supplementation on the gonads of male-to-female transgendered people. Testosterone is important in promoting proper testicular development, regulating both somatic and germ cells. Fetal exposure to high levels of testosterone can cause 46,XX DSD (CAH), whereas the androgen insensitivity can cause 46,XY DSD (AIS) (Table 1) [69]. Single-cell RNA sequencing has been performed on testis samples of two adult (46,XY) male-to-female transgendered people that were previously subjected to long-term testosterone reduction treatment using testosterone antagonists and estradiol [115]. When compared with untreated male testis samples, there was a lower proportion of differentiating spermatogonia (spermatocytes or spermatids). In contrast, the spermatogonia expressed undifferentiated markers. The 46,XY transgender female Sertoli cells were not similar to adult Sertoli cells, but to pubertal/juvenile Sertoli cells (11–13 yo), with higher levels of *AMH* and *HES1* mRNA [115]. These results reinforce the role of testosterone in spermatogonial differentiation and Sertoli cell maintenance, and it is intriguing that the Sertoli cells from the transgendered individual were different to those of an age-matched typical male. While DSD gonads are yet to be studied at single-cell resolution, this research demonstrates the potential of single-cell -omics for studying reproductive variations. This type of analysis will have an impact in developing new transgender hormone therapies and could be extended to hormonally derived DSD.

For individuals with DSDs at the gonadal level and with unknown molecular etiology, tissue biopsies could potentially be subjected to spatial transcriptomics, at near single-cell resolution. (Figure 5). This methodology may be useful in identifying the aberrant cell types involved and their transcriptional signatures, refining diagnosis and improving our understanding of how gene mutations can lead to DSDs. Another area in which single-cell approaches may be useful is in the generation and analysis of gonadal organoids. Organoids are artificial organs grown in culture, using knowledge of key regulatory genes expressed during specific organ development [181]. Organoids can be derived from induced pluripotent stem cells (iPS cells) and can be used to model normal and abnormal development. For the gonad, iPS cells have been used to generate Sertoli-like cells, and also Leydig and germ cells [182,183,184,185]. Single-cell RNA-seq could be used to chart the differentiation of these cell types, identifying important genes activated during formation of the testis. The effects of targeted gene deletion could then be examined in these organoids to model DSDs. 

### 7.2. Detection of Infertility by Single-Cell Sequencing

Around 7% of the male population suffers from infertility, a complex disease with a heterogeneous phenotype [186]. Similar to DSD, next generation sequencing technologies have improved the detection of putative genetic mutations associated with infertility, but the exact link between genotype and phenotype is unclear for most cases. Rodent models are still the gold standard used to correlate the genotype and phenotype in male (in)fertility [187]. Single-cell -omics applied to mouse models has been used to efficiently characterize the cellular and molecular bases of male and female infertility.

Single-cell RNA sequencing was used to study the testicular cell composition across mutant mouse strains (*Mlh3, Cul4a, Hormad1, Cnp* gene knockouts), all showing spermatogenesis defects. These were compared with wild type mice [175]. *Mlh3* and *Hormad1* knock outs showed complete early meiotic arrest, specifically in the mid-pachytene and leptotene/zygotene stages respectively, resulting in the absence of spermatozoa. The *Cul4a* mutant showed abnormal spermatids and a reduction in the post meiotic cells. *Cnp* null mice showed a defect in spermatogenesis, with a large reduction in the elongating spermatids. In addition, this research identified telocytes in the mouse testis, a novel interstitial cell [175]. In *Akap4* knockout mice subjected to scRNA-seq, an abnormal proportion of elongating spermatids was uncovered, with a reduction in expression of the key histone-associated kinase, Haspin and cytoskeleton/cilia assembly coiled-domain factor, Ccdc38 [174]. Single-cell -omics has demonstrated an extraordinary capacity to not only detect the precise transcriptomic changes associated with the different mutations, but to determine the cell stage where spermatogenesis had arrested. Such data is useful in developing efficient assisted reproductive techniques.

One recent study used scRNA-seq to explore the effects of deleting a single gene at a specific stage of spermatogenesis. *Sox30* was identified by single-cell transcriptomics to be highly expressed in mouse pachytene spermatocytes and round spermatids [120]. *Sox30* global and conditional knock out mice are sterile, lacking elongating and elongated spermatids but show the presence of multinucleated cells (arrested spermatids). Single-cell RNA-seq was then performed in *Sox30* null round spermatids, identifying more than 2000 differentially expressed genes compared to the wild type [120]. This research not only demonstrated the role of a novel regulator in spermatid development but also the potential of the single-cell -omics in detecting novel cell specific regulators.

Lacking sperm in the ejaculate is characteristic of non-obstructive azoospermia (NOA), the most severe form of male infertility [188]. Around 5–10% of the men evaluated for infertility have azoospermia due to defective spermatogenesis [189]. Single-cell RNA sequencing of a non-obstructive azoospermia patient was performed but no germ cells were identified among the sequenced cells [124]. Instead, Sertoli cells and a mix of peritubular myoid and Leydig cells were found. In addition, differentially expressed genes were found among the NOA patient cells versus fertile controls, largely associated with oxidative stress and DNA damage [124]. Further analysis of more NOA patients is necessary to identify critical spermatogenesis stages affected in this disease, which might result in new methodologies to restore fertility.

Single-cell approaches have also been applied to mouse models of ovarian dysfunction. In the ovary, progesterone receptor (Pgr) is essential for successful ovulation. Mice lacking *Pgr* expression in granulosa cells (*Esr2-PgrKO* mice) are infertile; they do not to release the oocyte during ovulation, resulting in oocytes present in corpora lutea [179]. Single-cell RNA sequencing was performed in wild type versus *Esr2-Pgr* KO ovaries. The mutant ovary contained double the number of immune cells than the wild type, consistent with the increased oxidative stress and hyper-inflammatory state that was detected [179]. Another ovarian disease that is highly associated with subfertility is Polycystic Ovary Syndrome (PCOS). Single-cell RNA-seq was used to identify the differences between oocytes and cumulus cells (granulosa cells adjacent to the oocyte in antral follicles) in healthy donors and PCOS patients undergoing assisted reproductive treatment (ART). PCOS cumulus cells from early stages of maturation (arrested oocyte in meiosis prophase) showed abnormal expression of genes involved in cell proliferation, hormone receptor signaling and oxidative stress, but were normal in cumulus cells from meiosis II metaphase oocytes [190]. Similar effects were found in the oocytes, with a down-regulation of hormone receptors in PCOS oocytes and dysfunctional meiosis maturation. Single-cell RNA-seq data showed that assisted reproductive techniques were able to promote ovulation and also repair poor quality oocytes in POCS patients and increase the incidence of fertilization [190]. All of these new research lines can be readily translated to the clinical environment, to improve the current ART and to explore causes of infertility. Interestingly, oxidative stress and cell death are common features in both male and female infertility revealed by scRNA-seq, making them an interesting target for future fertility treatments.

### 7.3. Single-Cell Sequencing in Cancer Biology 

Cancer results as a dysregulation in cell proliferation, differentiation and migration, key mechanisms also involved in embryogenesis [191]. It is not surprising that key developmental pathways are dysregulated in cancer progression, such as the Wnt, Notch and Hedgehog pathways. Understanding the role of these cancer associated genes and their involvement during normal gonadal development is critical to improve the diagnosis, prognosis and management of gonadal cancers.

Tumors are typically composed by a heterogeneous group of cells with different genetic and phenotypic characteristics, which involves not only cancer cells but also non-malignant cells, such as fibroblast, endothelial and immune cells [192]. Cancer displays not only transcriptomic but also genomic and karyotypic differences, which are important for understanding tumor potency and for designing specific treatments to delete malignant cells. Single-cell sequencing is ideally suited to understanding cancer biology, given the often heterogeneous nature of tumors. It allows the characterization of different cells present in the tumor, affording a better understanding of tumor composition and the interaction between cancer cells and surrounding tissues.

Single-cell genomics has been used to study the heterogeneous karyotypes and chromosome rearrangements present in human ovarian cancer samples. It has been used to detect whole chromosome aneuploidies, monosomies or tetrasomies, as well as rearranged chromosomes and focal amplifications among specific cell populations [178]. Diverse rearrangements have been found between and within different ovarian cancer samples, validated by multicolor fluorescent in situ hybridization (M-FISH) [178]. Single-cell transcriptomics has also been applied to high-grade serous ovarian cancer primary tumors and patient-derived xenograft models. Different cell types were identified in the tumors, including epithelial cells, cancer associated fibroblasts, macrophages, dendritic, B and T cells [177]. In addition, inter-patient heterogeneity was found in cancer cells (different subpopulations), but not in macrophages and fibroblasts. Single-cell RNA-seq information has also been used to detect chromosomal copy number alterations in malignant epithelial cancer cells [177], single nucleotide variants among cancer cells and can predict chemoresistance or estradiol responsiveness [172,173]. In contrast, testicular cancer single-cell transcriptomics is yet to be studied in detail. This applies to both stromal and germ cell tumors [193]. Single-cell sequencing can be a key tool to understanding the cellular complexity in heterogeneous tumors and predicting the most appropriate treatment. This will result in a transition to a more personalized therapy, focused on targeting specific cell types individually or simultaneously and eventually improving patient outcomes.

## 8. Considerations

Single-cell RNA-seq is providing deeper insights into normal gonadal development, gonadal maturation and disease, the germ cell niche and infertility. It’s application to DSDs is in its infancy. In order to use single-cell -omics in the clinic as a diagnostic tool for DSD it is necessary to generate a standardized protocol that can generate reproducible results with minimal artefacts. To achieve this aim, several aspects need to be taken into account, from the sample processing to the final bioinformatic analysis. The first major issue is obtaining gonadal samples from DSD patients. In DSD, the risk of malignancy is variable and in order to obtain an accurate risk assessment, a histological analysis of gonadal biopsy or gonadectomy is the recommended procedure [194]. Gonadectomy should only be performed in patients that have higher risks of developing malignant tumors. In “male-raised” individuals with mixed gonadal dysgenesis and in females with gonadal dysgenesis and Y chromosome material, a bilateral gonadectomy can be performed during early childhood. In contrast, the testes are typically removed during adulthood in “female-raised” individuals with complete or partial androgen insensitivity syndrome (46,XY DSD-AIS) and for “female-raised” individuals with defects in the androgen biosynthetic pathway (CAH), gonadectomy is sometimes performed before puberty [8,195]. Single-cell RNA-seq conducted in such tissues has the potential to shed new light on the mechanisms and manifestations of DSDs. However, as noted above, surgical removal of the gonads in DSD patients is bioethically fraught, as a significant school of thought considers it a violation of human rights [75,196,197]. 

In other settings, gonadal surgery can be considered as an option for fertility preservation. Most of women with Turner syndrome have high risk of developing primary ovarian insufficiency and infertility. Optional practices for fertility preservation in these cases are ovarian stimulation and later oocyte retrieval or ovarian tissue cryopreservation [198,199]. In individuals with Klinefelter syndrome, testicular sperm extraction by sampling a small portion of testicular tissue to isolate any viable sperm cell is possible, showing positive results. Meanwhile, testicular cryopreservation can be an option in prepubertal boys to obtain spermatogonia stem cells for future artificial differentiation and maturation [198]. Individuals with ovo-testicular DSD present both ovarian and testicular tissues in their gonads. The ovarian component tends to be histologically normal, whereas the testicular is often dysgenic, with limited numbers of germ cells and spermatozoa. Ovarian stimulation and oocyte retrieval or ovarian tissue cryopreservation are often the main options for these cases [198,199]. Knowledge of gene expression, growth factors and cell differentiation pathways gleaned from single-cell RNA-seq studies may inform efforts to preserve germ cells in these circumstances.

The advantage of scRNA-seq when conducted on gonadal biopsies, or on entire excised gonads, is that few cells are required (thousands). Gonadal samples from individuals with DSD or gonadal cancers can also be subjected to spatial transcriptomics, as outlined in Figure 5. Practical considerations include the fact that fresh samples are the best source for single-cell -omics, but this is not always a reality when dealing with clinical samples and, in most cases, they cannot be processed immediately. In order to maintain intact tissue, preservation methods should be developed to avoid cell death. Each tissue and cell type responds differently to diverse preservation methods, therefore ovarian and testicular tissues should be tested to identify the most suitable method to be used in the clinic [200]. In addition, sample storage time should also be considered to avoid any artefact in the analysis. Among the preservation methods, methanol fixation and cryopreservation are the most promising, being either performed in small tissue portions or on single-cell suspensions [200]. For spatial transcriptomics, fresh frozen tissue sections are required in order to maintain intact mRNA. Clinical samples are usually formalin fixed and paraffin embedded for longer preservation and maintenance of the tissue structure but often affecting mRNA quality. Recently, a successful methodology employed formalin fixed, paraffin embedded samples to obtain mouse brain mRNA spatial analysis [201]. New techniques are being developed to couple single-cell technologies to the clinical environment. Another key point in sample processing is the step used to obtain a single-cell suspension from a tissue sample, as in the case of the ovary or testis. Recently, it was shown that enzymatic digestion at 37 °C, although a typical methodology used nowadays, can induce cell death in specific cell populations, impacting single-cell analysis [200,202]. Endothelial and immune cells were found to be the most temperature-sensitive cell types [200]. New methodologies, such a cold dissociation protocols, are being developed to address this problem [200,202]. 

As noted above, two main technologies are currently being used for scRNA-seq, Smart-seq2 (plate based), allowing more sequencing depth but detecting less cells, and 10× genomics Chromium (droplet based), capturing more cells but at a lower sequencing depth [99]. Depending on the aims of the clinical diagnostic, one or the other can be used. If the gonadal composition and differential expression among the different cell types is more important, then Chromium technologies is best suited. However, if the sequencing depth is more important, for example, to detect lowly expressed genes, SNP or CNV, then Smart-seq2 is typically preferred. 

Lastly, one must consider the costs of these technologies. Single-cell -omics are more expensive that the current techniques used in diagnosis of DSD, infertility and gonadal cancers. Utilization of RNA-seq as the first line of diagnosis is currently not cost effective as a routine screening tool. Single-cell technologies should be applied, in principle, only to those cases where the definitive DSD etiology was not found or in cases where a genotype-phenotype correlation is required, with an explorative scope. It is expected that, as for bulk -omics technologies, the price of single-cell -omics reduce over time, making it accessible to a clinical environment. In addition, the use of Cell Hashing to pool different samples together will result in a reduction of the sequencing price per patient [110]. Single-cell -omics is a new area that is growing exponentially. We can expect new variations and technologies emerging in the following years that will contribute to developing an accessible and cost-efficient single-cell sequencing method that can be applied to clinical diagnosis of DSD, gonadal cancers and infertility. 

## 9. Conclusions and Future Perspectives

Single-cell sequencing technologies have demonstrated a large potential in characterizing the different cell types present in the ovary and testis during normal development and disease. Moreover, this approach can identify transcriptomic changes in a wide range of gonadal conditions including infertility and cancer, and potentially DSD. In comparison with the methodologies currently used in DSD diagnosis, single-cell -omics provides genetic and phenotypic information that can help to assess the risks associated with gonadal disease. It also has the potential to identify new personalized treatments or management strategies for DSD. The requirement of small number of cells for the analysis, together with the ability to detect simultaneously the transcriptomic, genomic and proteomic profiles of each individual cell makes single-cell multiomics an attractive technology for clinical settings. While single-cell -omics are not available for clinical diagnosis at the moment, we can speculate that they will contribute to the DSD diagnosis in the future. 

In the coming years, a standardized protocol should be developed in order to avoid cell loss during the gonadal sample storage and processing. This will also allow reproducibility of the diagnosis among different clinical centers. As noted above, different testicular and ovarian single-cell analyses have been performed at different developmental stages, but current reference samples are incomplete. Human testicular and ovarian atlases should be developed to understand the healthy development, differentiation and maturation of the gonads, at a refined single-cell level. The Human Cell Atlas project [111] will be helpful in achieving this goal. Once generated, these reference samples could be used in the clinic to compare a patient’s material, improving the diagnosis and helping develop new treatments. 

Improvements to the current single-cell technologies will no doubt advance, and automation will be introduced, allowing a more simple, powerful and cost-effective methodology to be used in both research lab and clinic. This also requires robust bioinformatics, relating the data back to the biology. This will result in a better understanding of typical gonadal development and conditions such as cancer, infertility and DSD.

## Figures and Tables

**Table 1:** Broad categories of Disorders/Differences in Sex Development (DSD) based upon type of origin, “gonadal” or “hormonal”.

Origin	46,XY DSD	46,XX DSD
DSD at the gonadal level	Gonadal dysgenesis (gonads are streaks of fibrous tissue) female Müllerian) ducts and variably feminized external genitalia Infertility	Gonads are testicular or partially testicular Male ducts Male or masculinized external genitalia Infertility
DSD at the hormonal level	Gonads typically testes Female ducts absent (AMH present) Partial or complete feminization of ducts and external genitalia due to androgen insensitivity (AIS)	Gonads typical ovaries Male ducts usually regressed External genitalia variably masculinized Elevated androgen levels, typically due to adrenal hyperplasia (CAH)
Methods of DSD diagnosis	-Prader scale for external genitalia -Ultrasound/laparoscopy of gonads and internal ducts -Karyotype analysis (46,XX, 46,XY, 46,X0, 46,XXY) -Biochemistry/endocrinology (e.g., steroid and peptide hormone levels) -Targeted gene mutation screening (e.g., SF1, SRY, CYP21A1, AR genes) -Whole genome or exome sequencing	

**Table 2:** Single-cell -omics research in gonadal development, differentiation and maturation.

Year	Authors	Organism	Developmental Stage	Tissue	Technology	Cells	Data
2015	Guo et al. [116]	Human embryo (male and female)	Males: 4w, 7w, 10w, 11w and 19w. Females: 4w, 8w, 10w, 11w and 17w	PGCs and somatic cells	MACS, FACS, Tang et al. [117]	319	GSE63818
2015	Lindeman et al. [118]	CAG-Stop-Dmrt1-Gfp; Sf1-Cre mice (female)	P17	GFP sorted supporting cells	FACS, SMARTer (C1)	68	GSE64960
2017	Li et al. [119]	Human embryo (male and female)	Males: 4w, 9w, 10w, 12w, 19w, 20w, 21w and 25w. Females: 5w, 7w, 8w, 10w, 11w, 12w, 14w, 18w, 20w, 23w, 24w and 26w	PGCs and somatic cells	MACS, FACS and Smart-seq2	2167	GSE86146
2018	Chen et al. [120]	Tg(Vasa-dTomato; Lin28-YFP) mice (male)	Unspecified	Spermatogenic cells	FACS, modified Smart-seq2	1136	GSE107644
2018	Green et al. [121]	C57BL/6J mice, Gfra1CreERT2; RosamT/mG, Amh-cre; RosamT/mG and Sox9-eGFP mice (male)	Adult	Whole testis, interstitial cells, spermatogonia and Sertoli cell enrichment, haploid depletion.	FACS, Drop-Seq	34,644	GSE112393
2018	Guo et al. [114]	Human (male)	Adult (17, 24 and 25 yo) and infant (13 months old)	Whole testis	Chromium	7790	GSE120508
2018	Hermann et al. [122]	Human and mice (male)	Human adults (mean = 41.6 yo). Mouse P6 and adults	Spermatogenic cells	FACS, Chromium & SMARTer (C1)	62,000	GSE108970, GSE108974, GSE108977, GSE109049, GSE109033 and GSE109037
2018	Stévant et al. [21]	Tg(Nr5a1-GFP) mice (male)	E10.5, E11.5, E12.5, E13.5 and E16.5	Nr5a1+ gonadal somatic cells	FACS, SMARTer (C1)	400	GSE97519
2018	Vértesy et al. [123]	Human (female)	8 to 14.4 weeks of development	Whole fetal gonads and adrenal germ cells	Smart-seq2	108	GSE79280
2018	Wang et al. [124]	Human adult (male)	Normal: 30 and 60 yo. Obstructive azoospermia: 27, 29, 34, 39, 41, 43 and 44 yo	Whole testis	Smart-seq2	2854	GSE106487
2018	Zhang et al. [125]	Human (female)	24 to 32yo	Oocytes and granulosa cells	Tang et al. [117]	151	GSE107746
2019	Ernst et al. [126]	C57BL/6J and TC1 mice (male)	P5, P10, P15, P20, P25, P30, P35, Adult (8–9 weeks)	Whole testis	Chromium	53,510	E-MTAB-6946
2019	Fan et al. [127]	Human (female)	Adult	Ovarian inner cortex	Chromium	20,676	GSE118127
2019	Grive et al. [128]	B6D2F1/J mice (male)	Postnatal days 6, 14, 18, 25, 30 and 8 weeks old	Whole testis, spermatogonia cells enrichment.	MACS, Chromium	15,493	GSE121904
2019	Gu et al. [129]	ICR Mouse (female)	14 days old	Mouse ES cells, MII oocytes and growing oocytes	scCOOL-seq [130] and Tang et al. [117]	1070	GSE114822
2019	Law et al. [131]	Blimp1-CreTg;Id4-eGFPTg;Rosa26tdTomato-fl_STOP_fl/LacZ mice (male)	E16.5, P0, P3 and P6	Germ cells	FACS, Chromium	10,140	GSE124904
2019	Sohni et al. [132]	Human (male)	Neonatal (2 and 7 days old) and adult (37 and 42 yo)	Whole testis	Chromium	33,585	GSE124263
2019	Stévant et al. [133]	Mouse Tg(Nr5a1-GFP) (female)	E10.5, E11.5, E12.5, E13.5, E16.5 and P6	Nr5a1+ gonadal somatic cells	FACS, SMARTer (C1)	563	GSE119766
2020	Estermann et al. [134]	Chicken embryo (male and female)	E4.5, E6.5, E8.5 and E10.5	Whole gonads	Chromium	33,247	GSE143337
2020	Guo et al. [115]	Human (male and transfemale)	Male: 7, 11, 13 and 14 yo. Transfemale: 26 and 50 yo	Whole testis	Chromium	9836	GSE134144
2020	Lau et al. [135]	Macaque (male)	Infant (1 yo), Juvenile (2 yo) and Adult (4 yo)	Whole testis and sorted spermatogonia cells	FACS, Chromium	16,932	E-MTAB-8979
2020	Niue et al. [34]	Mice (female)	E11.5, E12.5, E14.5, E16.5, E18.5, P1 and P5	Whole Ovary	Chromium	52,542	GSE136441
2020	Shami et al. [136]	Human and Macaque (male)	Adult human (20–40 yo), Adult Macaque (4–13 yo)	Whole testis	Drop-seq	36,000	GSE142585
2020	Tan et al. [137]	C57BL/6 mice (male)	E18.5, P2 and P7	Whole testis	Chromium	50,859	GSE130593
2020	Wagner et al. [138]	Human adult (female)	20–38 yo	Ovarian cortex and DDX4 sorted cell	FACS, Chromium and Smart-seq2	24,329	E-MTAb−8381, E-MTAb−8403
2020	Wang et al. [139]	Macaque (female)	Juvenile (4–5 yo) and Aged (18–20 yo)	Whole ovaries	STRT-seq	2601	GSE130664
2020	Xia et al. [140]	C57BL/6J mice and human (male)	Human adult (40 and 45 yo); 4 months old mice	Whole testis, germ cell enrichment	InDrops	4147	GSE125372

Abbreviations: E: embryonic day, P: postnatal day, w: weeks, yo: years old.

**Table 3:** Single-cell -omics research in gonadal disease, DSD, infertility and cancer.

Year	Authors	Organism	Developmental Stage	Tissue	Technology	Cells	Data
2016	Liu et al. [171]	Human (female) with or without POCS	Non-POCS (28.5 ± 3.75 yo). POCS (27.4 ± 3.02 yo)	Oocytes and cumulus cells	Smart-seq2	48	Unspecified
2017	Winterhoff et al. [172]	Human High-grade serous ovarian cancer (female)	Unspecified	Non-immune tumor cells (epithelial and stromal)	FACS, SMARTer (C1)	66	Unspecified
2018	Chen et al. [120]	Sox30tm1a(KOMP)Wtsi mice (male)	Adult	Spermatogenic cells	FACS sorted, modified Smart-seq2	85	GSE107644
2018	Vuong et al. [173]	FVB/N mice (female)	Unspecified	Ovarian surface epithelium cells, untreated or treated with estradiol	SMARTer (C1)	589	GSE121957
2018	Wang et al. [124]	Human adult (male)	Nonobstructive azoospermia: 24 yo	Whole testis	Smart-seq2	174	GSE106487
2019	Fang et al. [174]	C57BL/6J and Akap4-KO mice (male)	26 weeks old	Whole testis	Chromium	6804	SRR9107534
2019	Jung et al. [175]	C57BL/6J, B6;CBA-Tg(Pou5f1-EGFP)2Mnn/J, C57BL/6J CNP-EGFP BAC-TRAP mice, Mlh3-/-, Hormad1-/-, Cul4-/- and C57BL/6J CNP eGFP BAC TRAP mice (male)	11 to 38 weeks old	Whole testis, spermatocytes and spermatids cells enrichment	FACS and Drop-seq	57,600	GSE113293
2019	Liu et al. [176]	Human male, female and Klinefelter syndrome	Adult	PBMCs	Chromium	24,439	GSE136353
2020	Guo et al. [115]	Human (male and Transfemale)	Male: 7, 11, 13 and 14 yo. Transfemale: 26 and 50 yo	Whole testis	Chromium	9836	GSE134144
2020	Izar et al. [177]	Human High-grade serous ovarian cancer (female)	Unspecified	High-grade serous ovarian cancer	FACS, Smart-seq2 and Chromium	35,957	GSE146026
2020	Nelson et al. [178]	Human Ovarian cancer and stromal cells (female)	43–81 yo	Cultured ovarian cancer and stromal cells (Biobank)	SMARTer (C1) and Chromium	Unspecified	E-MTAB-8559
2020	Park et al. [179]	C57BL/6 and Esr2-PgrKO mice (female)	Unspecified	Whole ovaries	Chromium	6421	GSE145107

Abbreviations: yo: years old.

## Abbreviations

17-OHP	17-hydroxy-progesterone
AMH	Anti-Müllerian Hormone
AR	Androgen Receptor
CAH	Congenital Adrenal Hyperplasia
CGH	Comparative Genomic Hybridization
CNV	Copy Number Variants
DSD	Disorders of Sex Development
E	Embryonic day
FACS	Fluorescence-Activated Cell Sorting
NGS	Next-Generation Sequencing
iPS cells	induced Pluripotent Stem cells
LH	Luteinzing Hormone
MACS	Magnetic Activated Cell Sorting
PBMCs	Peripheral Blood Mononuclear cells
P	Postnatal Day
PCOS	Polycystic Ovary Syndrome
scRNA-seq	Single-Cell RNA Sequencing
SNP	Single Nucleotide Polymorphisms
t-SNE	t-distributed Stochastic Neighbor Embedding
UMAP	Uniform Manifold Approximation and Projection
yo	Years Old
